# FieldTrip Made Easy: An Analysis Protocol for Group Analysis of the Auditory Steady State Brain Response in Time, Frequency, and Space

**DOI:** 10.3389/fnins.2018.00711

**Published:** 2018-10-09

**Authors:** Tzvetan Popov, Robert Oostenveld, Jan M. Schoffelen

**Affiliations:** ^1^Department of Psychology, University of Konstanz, Konstanz, Germany; ^2^Donders Institute for Brain, Cognition and Behaviour, Radboud University, Nijmegen, Netherlands; ^3^NatMEG, Department of Clinical Neuroscience, Karolinska Institutet, Stockholm, Sweden

**Keywords:** ASSR, EEG, FieldTrip, group analysis, ERP, beamforming

## Abstract

The auditory steady state evoked response (ASSR) is a robust and frequently utilized phenomenon in psychophysiological research. It reflects the auditory cortical response to an amplitude-modulated constant carrier frequency signal. The present report provides a concrete example of a group analysis of the EEG data from 29 healthy human participants, recorded during an ASSR paradigm, using the FieldTrip toolbox. First, we demonstrate sensor-level analysis in the time domain, allowing for a description of the event-related potentials (ERPs), as well as their statistical evaluation. Second, frequency analysis is applied to describe the spectral characteristics of the ASSR, followed by group level statistical analysis in the frequency domain. Third, we show how time- and frequency-domain analysis approaches can be combined in order to describe the temporal and spectral development of the ASSR. Finally, we demonstrate source reconstruction techniques to characterize the primary neural generators of the ASSR. Throughout, we pay special attention to explaining the design of the analysis pipeline for single subjects and for the group level analysis. The pipeline presented here can be adjusted to accommodate other experimental paradigms and may serve as a template for similar analyses.

## Introduction

Multi-subject clinical or cognitive studies that require the group analysis of large amounts of electrophysiological data may be challenging for the researcher involved, for several reasons. For instance, although many software packages for electrophysiological analysis have well documented functionality for individual subject analysis, the possibilities for group analysis are limited, requiring the researcher to export single subject values into a generic statistical package such as SPSS. In addition, many EEG system specific software packages (e.g., BrainVision Analyzer and Neuroscan Scan), as well as many commercially available generic packages (e.g., ASA, BESA, and Curry), require the user to interact with the data through a graphical user interface (GUI). Although the GUI allows for easy visualization and interaction with the data, the execution of error free, consistent (across subjects), and reproducible analysis protocols requires the researcher to be meticulous during the interaction with the GUI. Also, GUI-based software does not provide an easy way to re-evaluate group-level effects after making changes to the analysis parameters. Finally, by nature of the graphical user interface, the researcher’s documentation of the analysis pipeline is often inadequate for robust reproducibility; in the best case the pipeline documentation comprises a descriptive recipe of which buttons to press, but often it only outlines the general idea of the analysis without sufficient details required to reproduce it.

Software tools that allow for researcher-data interactions by means of scripts provide some advantages to purely GUI-based software, with respect to the ease with which important aspects of the data analysis protocol can be addressed. Consistent single-subject analysis is ensured by (re-)using the exact same sequence of commands across subjects. Pipelines can be easily rerun, which facilitates the evaluation of parameter choices. Also, reproducibility of analysis results can be enhanced by publishing or sharing the scripts. FieldTrip (Oostenveld et al., 2011) is a well-established open source MATLAB toolbox for script-based electrophysiological data analysis. FieldTrip does not have a GUI and requires some programming proficiency from its users in order to write and evaluate scripts; this may deter researchers from using it for their data analysis, despite the advantages from script-based analysis as outlined above.

**FIGURE 1:**
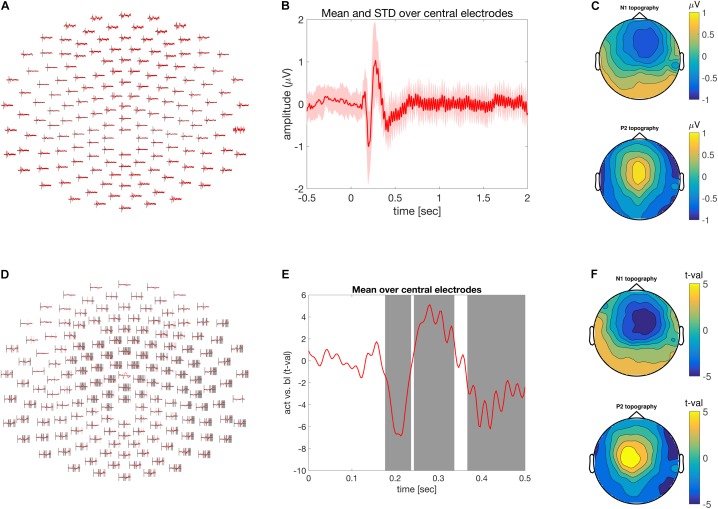
Time-domain analysis. **(A)** Grand average of the ASSR across multiple electrodes. **(B)** ASSR averaged across central electrodes. Time is depicted on the abscissa and amplitude on the ordinate. **(C)** Scalp topography of the N1 top (170–230 ms) and P2 bottom (250–300) components of the grand averaged ASSR. **(D)** Difference between pre and post-stimulus activity across multiple electrodes expressed as units of *t*-values. Shaded areas at electrode clusters reflect time clusters motivating the rejection of the null hypothesis. **(E)** Time course of the difference between pre and post-stimulus activity expressed in units of *t*-values. Gray areas highlight the time clusters of significant condition differences. Time is depicted on the x-axis and difference strength on the y-axis. **(F)** Similar to **(C)**, but in units of *t*-values.

In this study we demonstrate how to perform group analysis of electroencephalographic data, from raw EEG to publishable visualization of results, using FieldTrip. It is primarily aimed at researchers with (yet) little technical know-how and demonstrates that such an analysis comprises a relatively limited amount of computer code to implement a meaningful pipeline. We provide step-by-step recipes, each of which implements a conceptual analysis step. The execution time of the whole sequence of analysis steps (e.g., resulting in **Figure 1**) is less than 30 min on a typical computer (MacBook Pro, 2.8 GHz, 16 GB RAM), and the organization of the code allows for fast re-computation and evaluation of results after changing some specific parameters. Documentation of the analysis pipeline is straightforward, since the scripts fully reflect the pipeline, thus fostering efficient re-evaluation of analysis protocols, reproducibility of findings and exchange between colleagues and research groups.

The data used in this study comes from an experiment that used the auditory steady state evoked response (ASSR), a robust and replicable phenomenon in psychophysiological research (Stapells et al., 1984). In a representative application, the amplitude of a carrier sinusoidal sound wave (e.g., at 500 Hz) is modulated by a periodically varying envelope (e.g., at 40 Hz). This amplitude-modulated (AM) stimulus evokes a clear 40 Hz auditory evoked potential in the electroencephalogram (EEG) or auditory evoked field in the magnetoencephalogram (MEG) (Legget et al., 2017). ASSR-paradigms have a widespread adoption in clinical studies (Korczak et al., 2012; O’Donnell et al., 2013), in patients with tinnitus (Wienbruch et al., 2006; Schlee et al., 2008; Diesch et al., 2010; Sereda et al., 2013), schizophrenia (Spencer et al., 2008; Krishnan et al., 2009; Rass et al., 2012), major depressive disorder (Chen et al., 2016; Isomura et al., 2016), and autism (Wilson et al., 2007; Edgar et al., 2016; Rojas et al., 2011). ASSR-paradigms are also used as a diagnostic tool to estimate normal and abnormal hearing sensitivity (Korczak et al., 2012). In cognitive studies, ASSR-paradigms have for example been used to demonstrate the functional tonotopic organization of the human auditory cortex (Pantev et al., 1996), and as means of evaluating the effects of top-down attention on early sensory processes (Ross et al., 2004; Muller et al., 2009; Weisz et al., 2012).

We provide step-by-step analysis for group level analysis, using the open source MATLAB toolbox FieldTrip. All scripts and data are available at https://doi.org/11633/di.dccn.DSC_3015000.00_810 to allow readers to reproduce the analyses in details. The first part will be focused on analysis in the time domain and focuses on the group-level characteristics of the stimulus-onset related ERP. In the second part, we will analyze the ERPs in the frequency domain, quantifying the steady-state response. We conclude the analysis pipeline by applying a spatial filtering algorithm to the time and frequency domain data in order to identify the cortical generators of the auditory evoked response.

## Materials and Methods

### Participants

Twenty-nine healthy individuals (11 female) participated in the study. The mean age was 28 years, ranging from 21 to 36 years. All participants gave written informed consent in accordance with the declaration of Helsinki. The study design and protocol were approved by the local Ethics Committee of the University of Konstanz. Inclusion criteria were: normal intellectual abilities, no history of psychiatric or neurological disorders with loss of consciousness and no substance abuse. Prior to experiment participants were screened with the Mini International Neuropsychiatric Interview (Sheehan et al., 1998). All but two participants were right handed as confirmed by the Edinburgh Handedness Inventory (Oldfield, 1971). Following participation, all subjects were compensated with 20 Euros.

### Stimuli and Experimental Task

Stimuli were similar to previous reports (Muller et al., 2009). We used a composite signal consisting of 500 Hz carrier sine wave modulated by a 40 Hz sinusoidal signal. This composite signal was presented with a sampling rate of 44.100 Hz and a modulation depth of 100%. At stimulus onset and offset we used a 50 ms fade in and fade out period, in order to avoid these to be audible as a clicking noise. A total of 120 epochs of 2 s were acquired with a 1 s inter-trial interval (ITI). All stimuli were applied via headphones to the left ear only, thus engaging predominantly right auditory cortical areas.

### Data Acquisition

EEG was acquired while the subjects were seated in an acoustically shielded room, using a high-density, 256-channel EGI system with a HydroCel Geodesic Sensor Net (Electrical Geodesics, Inc., Eugene, OR, United States). Data was filtered with 0.1 Hz high-pass and 400 Hz low-pass hardware filters and sampled at 1 KHz. The vertex (Cz) electrode served as reference during data recording. In line with EGI acquisition guidelines, the electrode impedances were kept below 30 kΩ. All of the reported analyses were performed on a MacBook Pro with a 2.8 GHz processor, 16 GB 1600 MHz DDR3 RAM and using MATLAB version 2017b (MathWorks, Natick, MA, United States).

### Data Epoching and General Preprocessing

In general, an analysis pipeline implemented using the FieldTrip toolbox consists of a sequence of calls to specific functions from the toolbox. Each of these functions reflect a conceptual analysis step and produce as an output a data object. The input to these functions is always a so-called configuration (cfg) structure, which specifies the options and algorithmic parameters used by the function, and usually the data is passed as second input argument. Here, the first step of our analysis pipeline consisted of the specification of occurrence of epochs-of-interest (trials) in the raw data file, based on the timing information of the stimulus onset triggers that were recorded along with the continuous data. This first step was followed by the reading in of the epochs-of-interest, followed by the application of a bandpass filter and rereferencing to the average reference. In FieldTrip, the extraction of the epochs is achieved with a call to the ***ft_definetrial*** function. Reading the data from disk and basic preprocessing is achieved with a call to the function ***ft_preprocessing***.

***Ft_definetrial*** is a function that extracts the event structure from a raw data file, for instance by reading a digital trigger channel, or by extracting timestamps of relevant events or annotations from the metadata. Next, the event structure is interpreted to define the onsets and offsets of epochs-of-interest. This requires the specification of the event values of interest, which in this specific dataset are the triggers ‘DIN3’ and ‘DIN5.’ Also, the cfg needs to contain information with respect to the length of the requested epochs, defined in seconds. In this case, we specified *cfg.trialdef.prestim = 1;* and *cfg.trialdef.poststim = 3*, i.e., 1 s before and 3 s after stimulus onset, to allow for a sufficient length for artifact-free digital filtering. Subsequently, using the epoch definition as obtained from ***ft_definetrial***, the function ***ft_preprocessing*** was used to read in the data from a specified set of channels, apply offline re-referencing to the average reference, and a digital bandpass filter. The relevant options specified were: (1) *cfg.channel = egi256customlay.label*, for the channel selection (egi256customlay is a MATLAB structure, the label field contains a list with the names of all EEG channels), (2) *cfg.reref = ‘yes*,*’* together with *cfg.refchannel = ‘all’* for the rereferencing, and (3) *cfg.bpfilter = ‘yes’; cfg.bpfreq = [1 48]; cfg.bpfilttype = ‘firws*,*’* for the filtering [a filter type of firws uses a windowed-sinc finite impulse response filter (Widmann et al., 2015)]. These analysis steps are documented in the analysis script *ASSR_timedomain.m* from line 1 to line 53. The required time to run this step was 19 min.

### Computation of Event-Related Potentials and of the Group Average

To compute the event-related potentials (ERPs) per subject, the epoched time series is averaged with the ***ft_timelockanalysis*** function. The grand mean ERP is obtained by including all individual subjects’ ERP data structures as an input argument to the ***ft_timelockgrandaverage*** function. In addition to computing an average ERP, these functions allow for the selection of specific latency windows and/or subsets of channels for subsequent analysis. Here, we used *cfg.latency = [-0.5 2];* and *cfg.channel = [‘all’ ‘-E30’ ‘-E192’];* to do a selection of the latency window, and to discard 2 bad electrodes. The computation of individual ERPs and the grand mean ERP took 8 min and is documented in *ASSR_timedomain.m* lines 58–84.

### Visualization of the Group Average ERP

FieldTrip contains several functions to visualize the spatiotemporal structure of EEG data. Here, we demonstrate the use of ***ft_multiplotER, ft_singleplotER***, and ***ft_topoplotER***, which display ERP-data, as a set of ERP time courses on a channel layout, an average time course across a set of specified channels, and a spatial topography in a specified latency window, respectively. This set of functions allows for interactive exploration of the data by making iterative selections (by dragging a square in the figure panel) of subsets of channels or latency windows for displaying time courses and topographies.

These functions require a layout of a 2-dimensional projection of the electrode positions on the computer screen. Most EEG recording systems do not represent electrode positions in the subjects’ data files; a generic way to deal with this is to specify a template layout in the *cfg.layout* field. The prerequisite here is that the electrode names in the layout file match the electrode names in the data structure’s channel-field. For these data, we have created a custom layout *‘egi256customlay.mat,’* which excludes the neck and cheek electrodes from the electrode array. Further information about the construction and design of custom layouts can be found in the layout tutorial and information about the available template layouts can be found in the template documentation^1^.

The call to the ***ft_multiplotER*** function will produce the illustration in **Figure 1A** (in *ASSR_timedomain.m*, lines 90–95). The auditory event related potential over fronto-central electrodes can be visualized using the function ***ft_singleplotER*** with the additional configuration options specifying the desired channels in *cfg.channel* and controlling the ordinate range between -1 and 1 mV in *cfg.ylim*. The color of the line is specified with *cfg.graphcolor* and the linewidth is controlled with *cfg.linewidth* (in *ASSR_timedomain.m*, lines 96–104). It is often useful to illustrate the level of variation across participants. This can be achieved by specifying *cfg.keepindividual = ‘yes’;* during the call to ***ft_timelockgrandaverage***. The activity of one or a group of electrodes can be selected and averaged using the function **ft_selectdata** and the configuration options *cfg.channel* and *cfg.avgoverchan*. Finally, using the MATLAB functions ‘patch’ and ‘line’ an averaged response together with its corresponding standard deviation can be visualized. This is illustrated in **Figure 1B** (in ‘ASSR_timedomain.m,’ lines 117–141).

A topographic illustration of the data is provided using the function ***ft_topoplotER*** focusing of the N100 (170 to 230 ms post-stimulus onset) and P200 (250 to 300 ms post-stimulus onset) components. The latency specification is provided by the configuration option *cfg.xlim*. The other configuration options pertain to the particular illustration style and depend on the taste of the user. Typing ‘help ft_topoplotER’ in the MATLAB command window gives a detailed overview of the various configuration alternatives. Lines 144–168 in *ASSR_timedomain.m* reproduce **Figure 1C**.

### Statistical Evaluation of Stimulus Onset Evoked Activity

In FieldTrip, statistical decisions can be done using non-parametric statistical tests using spatiotemporal clustering to address the family-wise error rate (Maris and Oostenveld, 2007). The general idea is that real neurophysiological effects have specific structure in the spatiotemporal data matrix, which can be exploited in order to maximize statistical sensitivity. Due to the temporal and spatial structure in the EEG signal, neighboring time-points and electrodes are likely to reflect the same neural phenomena; aggregating these spatiotemporal neighbors into clusters pools the evidence for an effect being present. The spatiotemporal clustering is combined with a permutation framework to generate a distribution of expected cluster-based test statistics under the null-hypothesis of exchangeability of the data across conditions or experimental groups. In this way, a large number of statistical evaluations, which is custom in a mass univariate context, is reduced to just a single statistical evaluation, thus elegantly providing control for the family-wise error rate. Cluster-based statistical testing provides a powerful approach to EEG and MEG data. We recommend this detailed overview with accessible examples for further reading^2^.

In order to be able to form clusters across the spatial dimension, a so-called neighborhood structure needs to be created. This structure contains information about the spatial distribution of the electrodes, and which electrodes are considered to be neighbors. For this purpose, we use the function ***ft_prepare_neighbours*** and store the output in a variable called ‘neighbors’ (in *ASSR_timedomain.m*, lines 174–181). In many EEG setups the exact geometrical information about the 3-D spatial distribution of the electrodes is not directly available from the data. To address this, FieldTrip’s template directory contains a collection of common electrode geometries. Here we read in the standard 3D template file of EGI’s 256 electrode net, using the function ***ft_read_sens***; the position of all electrodes is subsequently used to determine the spatial neighbors of each electrode.

In a typical experimental context, statistical inference is done on a contrast between experimental conditions or groups. To illustrate a group-level statistical evaluation in the data presented here, where we just have a single condition, we will compare the early post-stimulus-induced signals with the pre-stimulus baseline window, where the length of the windows is matched. To this end, we first split the single participant ERP data into 2 data structures, representing the baseline and the post-stimulus onset segments, respectively. This can be achieved with the function ***ft_selectdata***, where the *cfg.latency* option specifies the latency window of the data that is to be cut out from the longer segments. The length of the data in both ‘conditions’ is kept equal, i.e., 500 ms to facilitate subsequent comparison (in *ASSR_timedomain.m*, lines 184–193). This step in our pipeline results in two variables ‘act’ and ‘bl,’ which are arrays that contain the single participant ERP data for the selected time segments. The statistical evaluation is performed by calling the function ***ft_timelockstatistics***, using a somewhat elaborate configuration in order to specify the exact details of the statistical evaluation procedure. For instance, it is also possible to perform regular parametric mass-univariate statistical inference, or to specify other multiple comparison correction schemes. Here, in order to apply non-parametric permutation based inference, we need to specify *cfg.method = ‘montecarlo.’* As a consequence, the function will provide a Monte Carlo approximation of the randomization distribution for a chosen test statistic, which is specified to be a dependent samples T-statistics (*cfg.statistic = ‘ft_statfun_depsamplesT’)*. In order to exploit the spatiotemporal clustering scheme for multiple comparisons correction, we specify *cfg.correctm = ‘cluster*.’ Once it is decided to use the clustering scheme for multiple comparison correction, a channel neighborhood structure needs to be provided, as indicated above, in the *cfg.neighbours* field. The sample specific T-statistic, 1000 samples of which as defined by *cfg.numrandomization = 1000;* are produced and form the randomization distribution of the test statistic. The probability of falsely rejecting the null hypothesis is defined by *cfg.alpha = 0.025;* corresponding to a false alarm rate of 0.05 divided by 2 in a two-sided test.

The final important piece of information that needs to be provided, is *cfg.design*, which is a FieldTrip style ‘design matrix’ that specifies how the individual input data objects relate to the experimental subjects and conditions. The code for the statistical evaluation is documented in *ASSR_timedomain.m* lines 197 to 222.

To visualize the output of the statistical evaluation, we can use the same functionality as for the visualization of the grand average ERP, as described above. With ***ft_multiplotER***, in combination with *cfg.parameter = ‘stat’* we can obtain a figure that displays the test-statistic as a function of time and electrode, in this case it shows *t*-values. We can also specify a *cfg.maskparameter*, which is the fieldname of the numeric data in the input structure that can be used to highlight specific spatiotemporal features in the data. In this case we used the ‘mask’-field, which highlights the time points that constitute the cluster of spatiotemporally contiguous data points, on the basis of which the null hypothesis is rejected. Lines 225 to 232 in *ASSR_timedomain.m* reproduces **Figure 1D**.

Using the function ***ft_singleplotER*** the time-course of condition differences can be illustrated for the set of fronto-central electrodes described above. Lines 235–249 in *ASSR_timedomain.m* generate the illustration in **Figure 1E**.

Finally, a topographic representation of the observed effect is visualized in the same manner as the topography of the N1 and P2 components, yet instead of scalp distribution of amplitude differences, a scalp distribution of condition difference expressed in *t*-values is shown in **Figure 1F** and lines 252–275 in *ASSR_timedomain.m*. The entire statistical evaluation and visualization of the outcome took 1 min and 45 s.

### Frequency Domain Analysis

Frequency analysis was performed on the individual ERP data and is documented in the analysis script *‘ASSR_freqdomain.m.’* This part of the pipeline aims to quantify the electrophysiological response to the actual steady-state stimulation. To this end, the trial-averaged ERP is spectrally decomposed, while focusing on a post-stimulus onset latency window, after the stimulus transients have subsided. This approach, i.e., performing a spectral analysis on trial-averaged time courses, is only optimal in situations where the phase of the carrier wave signal is identical across trials. If this is not the case, trial-based averaging in the time domain leads to cancelation effects, and thus to suboptimal estimates of the steady-state response if the spectral analysis is performed on these averages. As an alternative, inter-trial coherence (ITC) can be also computed. Individual ERP’s are loaded and data of equal length (1 sec) during baseline and post-stimulus onset was extracted (lines 34 to 39 in *ASSR_freqdomain.m*).

Subsequently, for each individual and condition (‘bl’ and ‘act’) power spectra were computed, based on fast Fourier transformation (FFT) of the segmented data after the application of a Hanning taper. This was done using the function ***ft_freqanalysis***, with *cfg.method = ‘mtmfft*,’ and *cfg.taper = ‘hanning*.*’* Frequencies of interest can be defined by the configuration option *cfg.foilim* and were specified in the range from 0 to 45 Hz (in *ASSR_freqdomain.m*, lines 42–48). A detailed overview of the various spectral decomposition methods, the rationale behind them as well as their application is documented in an online tutorial^3^.

Following spectral decomposition, a grand averaged frequency domain activity per condition was computed using the function ***ft_freqgrandaverage***.

**FIGURE 2:**
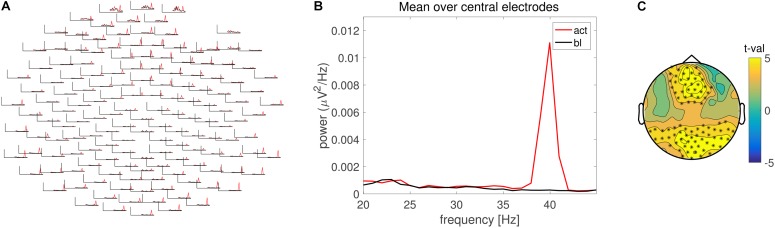
Frequency domain analysis. **(A)** Grand average power spectrum for the pre- (black) and post-stimulus (red) ASSR across multiple electrodes. **(B)** Grand average power spectrum averaged across central electrodes. Line color identical to **(A)**. **(C)** Scalp topography of the condition difference in 40 Hz power expressed in units of *t*-values. Electrode clusters on the basis of which the null hypothesis was rejected are highlighted with asterisks.

Visualization of the grand averaged spectra can be illustrated with the function ***ft_mulitplotER***, much in the same way as for the time domain data. Lines 60 to 66 in *ASSR_freqdomain.m* reproduce the illustration in **Figure 2A**. ***Ft_singleplotER*** can be used to visualize the spectrum, averaged across sets of electrodes. Note that in contrast to the time domain illustration above, two input arguments are used. In this way, the spectral content of the data during the baseline and the stimulation activity can be visualized in a single figure (**Figure 2B**, lines 69–78 in *ASSR_freqdomain.m*). The total duration of frequency analysis is 30 s.

### Statistical Evaluation of Frequency Domain Data

Statistical evaluation of the difference in spectral power between conditions can be examined using the function ***ft_freqstatistics***. The configuration options remain largely the same, as compared to the time domain comparison described above. Because the research question pertains to a particular frequency (i.e., 40 Hz response) the statistical evaluation is constrained to a ±1 Hz around this frequency by the configuration options *cfg.frequency* and *cfg.avgoverfreq*. For didactical reasons we constrain the threshold of *cfg.clusteralpha*.

The outcome of the statistical evaluation can again be illustrated with the function ***ft_topoplotER***. The electrodes contributing to the spatial cluster on the basis of which significant condition differences were found can be visualized by the option *cfg.highlight* in combination with *cfg.highlightchannel.* These channels were defined using the binary mask provided in the structure ‘*statfreq*.’ Lines 93 to 138 in ‘*ASSR_freqdomain.m*’ reproduce **Figure 2C**. Duration of statistical evaluation of frequency domain data is 16 s.

### Time-Frequency Domain Analyses

**FIGURE 3:**
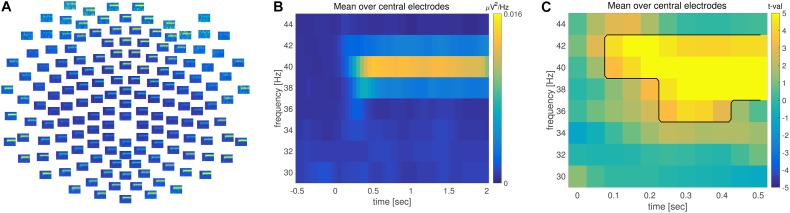
Time-frequency analysis. **(A)** Grand averaged time-frequency representation of power (TFR) across multiple electrodes. **(B)** TFR averaged across central electrodes. Time is depicted on the x and frequency on the y axis. Warm colors reflect increase of oscillatory power. **(C)** TFR of the difference between pre and post-stimulus activity. Axis are identical to **(B)**; warm colors correspond to an increase and cold colors to a decrease in power during post-relative to pre-stimulus. The observed time-frequency cluster on the basis of which the null hypothesis is rejected is highlighted by the black line.

In a typical application, spectral analysis techniques are used to obtain a time-frequency representation (TFR) of the signals. In FieldTrip, this decomposition is done with the function ***ft_freqanalysis***, specifying one of the supported time-frequency decomposition algorithms in *cfg.method*. Here, we demonstrate this functionality in the analysis script *‘ASSR_timefreqdomain*,’ using the method ‘mtmconvol,’ which implements a sliding window FFT. A window of 500 ms was used that slid over the data with increments of 50 ms. The desired output of this analysis was spectral power thus *cfg.output = ‘pow,’* using convolution in the frequency domain specified by *cfg.method = ‘mtmconvol.’* The frequency bins of interest are defined by *cfg.foi* and the time points of interest by the option *cfg.toi*. The length of the time window per frequency of interest is defined by *cfg.t_ftimwin*. Here, we used as input signals the individual trial-averaged ERPs. Note that in typical applications where the induced activity is time-locked but not phase-locked to the onset of an event, single trial data should be entered in the analysis. Following grand averaging using the ***ft_freqgrandaverage*** the output can be visualized using the function ***ft_multiplotTFR***. Lines 24–64 in *ASSR_timefreqdomain.m* reproduce **Figure 3A** and require 140 s computing time.

The average of the time-frequency representation of power (TFR) over the fronto-central electrodes can be illustrated using the function ***ft_singleplotTFR*** lines 68–75 in *ASSR_timefreqdomain.m*.

### Statistical Evaluation of Time-Frequency Data

Similar to time and frequency domain data, statistical evaluation of time-frequency data can be achieved by the function ***ft_freqstatistics***. This is documented in lines 91–155 in *ASSR_timefreqdomain.m* and is computed within 57 s.

### Reconstruction of Evoked Potentials and the Steady-State Response With Beamforming

#### Time Domain Beamforming

Source reconstruction of neuronal activity can be performed using various algorithmic approaches. FieldTrip contains functionality for parametric dipole modeling (using the function ***ft_dipolefitting***), distributed source modeling (minimum-norm estimation), and beamformers (using the function ***ft_sourceanalysis***). Here, we demonstrate source reconstruction with beamformers. First, we used a linearly constrained minimum variance (LCMV) beamformer (Van Veen et al., 1997), to reconstruct the ERP at the cortical level. Beamformer methods use the data covariance matrix and forward models that are specific to the locations-of-interest to construct spatial filters optimized for these locations. Beamformers are often applied by scanning the whole brain, computing the spatial filter for each location. Ideally, forward models are computed using volume conduction models based on individual participants’ anatomical information, yet this information is often not available for participants to EEG studies. In that case, forward models can be computed using template volume conduction models, in combination with a specification of the electrode positions, coregistered to the conduction model. Here, the forward model was calculated using a realistically shaped three-layer boundary-element volume conduction model, on a 3-dimensional grid of dipole locations with equidistant spacing of 8 mm.

The procedures are documented in the analysis script *‘ASSR_lcmv.m.’* This analysis in source space is focused on the stimulus onset response, and particularly the N1 latency window from 170 to 230 ms post-stimulus onset (see above). First, the leadfield, the volume conduction model of the head (i.e., the headmodel), as well as the position of the electrodes are loaded into memory and visualized (lines 28–40, in ‘*ASSR_lcmv.m*’). Subsequently, for each subject the epoched data is loaded where the preparation of the sensor-level data largely follows the same recipe as the ERP analysis. The most important difference with the earlier procedure is that the source analysis requires an estimate of the sensor covariance matrix of the epoched (yet unaveraged) data. Bad electrodes are excluded using ***ft_selectdata*** and the data is bandpass filtered between 1 and 40 Hz using ***ft_preprocessing***. The data covariance is estimated using the function ***ft_timelockanalysis*** (lines 63–67 in ‘*ASSR_lcmv.m*’). The data consists of fewer channels than the precomputed leadfield. The section in *ASSR_lcmv.m* (lines 71–80) prunes the leadfields as pre channels present in the data.

Source analysis is performed using the function ***ft_sourceanalysis***. The desired reconstruction method is defined by the option *cfg.method = ‘lcmv’*; in combination with a specification of the electrodes (*cfg.channel*), leadfield (*cfg.grid*) and headmodel (*cfg.headmodel*). Additional options, specific to the *lcmv* method, can be defined by *cfg.lcmv.xxx.* Here, we included the preservation of the spatial filters in the output structure (*cfg.lcmv.keepfilter*), a fixed dipole orientation (*cfg.lcmv.fixedori*), weights normalization accounting for the center of the head bias (*cfg.lcmv.weightnorm*) together with (*cfg.lcmv.projectnoise*), and regularization parameter (*cfg.lcmv.lambda*). This is documented in ‘*ASSR_lcmv.m*’ lines 83–110.

A more detailed description of source analysis strategies and their implementation can be found in a series of online tutorials^4^. The total duration of source analysis in the time–domain is 16 min.

**FIGURE 4:**
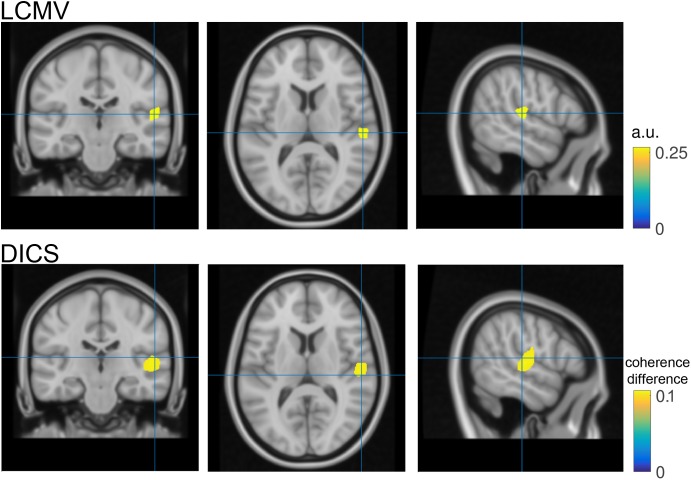
Source reconstruction. Top- Coronal, transversal and axial slices of reconstructed N1 activity in neurological convention. The blue cross hairs correspond to the maximal activation within the right Heschl’s gyrus. Bottom- same as top but utilizing dynamic imaging of coherent sources (DICS) as source reconstruction method. Color code represents the increase in coherence with a surrogate 40 Hz signal during the post-stimulation period as compared to the pre-stimulus baseline.

To visualize the outcome of the source analysis, the individual source reconstructions are loaded and averaged across subjects, to obtain the grand-average of brain activity during the N1 latency window (**Figure 4** top row). This is done by the function ***ft_sourcegrandaverage*** and the result can be interpolated onto a template anatomical MRI using ***ft_sourceinterpolate*** (lines 127–135, in *ASSR_lcmv.m*). A volumetric atlas according to Automated Anatomical Labeling (AAL) scheme (Tzourio-Mazoyer et al., 2002) can be read with the function ***ft_read_atlas*** (line 138 in *ASSR_lcmv.m*). Finally, the interpolated grand-average is visualized using the function ***ft_sourceplot***, where the anatomical information about activity at a given location is specified by the *cfg.atlas = aal;* option. In addition, a thresholding mask is created to highlight 98% of maximum activity. This mask is applied during the plotting if the *cfg.maskparameter* option has been specified (lines 139–148 in *ASSR_lcmv.m*). The computational time of grand averaging and visualization is 2 min.

#### Frequency Domain Beamforming

Rather then focusing on a particular latency, it is also possible to reconstruct activity for a given frequency of interest. The utilized stimulation frequency of 40 Hz appears an appropriate target for frequency domain beamforming. Here, we demonstrate the use of Dynamic Imaging of Coherent Sources (DICS) (Gross et al., 2001), and source reconstruct the cortical response that is phase-locked to the 40 Hz modulation signal by means of the coherence coefficient. In the current context of steady-state auditory stimulation, this procedure turns out to be a bit more involved than a straightforward sequence of FieldTrip functions in their standard application, and requires some additional manipulations to the data to work. The reason for this is that steady-state auditory stimulation induces a high SNR signal that leads to (near) simultaneous (i.e., zero-lag correlated) activation in bilateral auditory areas. Consequently, traditional beamformers (which scan the brain using a single equivalent current dipole as the underlying source model) will fail to yield reliable results, because of the violation of the underlying assumption that for any source-of-interest there are no other temporally correlated sources. To address this issue, more sophisticated beamformer source models can be used (Dalal et al., 2008; Schoffelen and Gross, 2009). In this case, we implemented the beamformer to scan through the brain volume, and reconstruct the activity of pairs of dipoles, where left/right symmetric dipole pairs were used as a source model.

The procedures are documented in the analysis script *‘ASSR_dics.m’* and are largely similar to the ones described above. The symmetric dipole source model is created on lines 41–50 in *ASSR_dics.m*. FieldTrip allows for the creation of symmetric dipole-pair source models in the function ft_prepare_sourcemodel, but in the current example we bypass this functionality, because we are working with precomputed leadfields. Alternatively, in the example script we create compound leadfields directly, by concatenating for each grid position, the corresponding dipole’s forward model, with the forward model in the opposite hemisphere. In addition, we create a ‘dummy’ signal that represents the 40 Hz modulation signal, which is needed for the coherence computation. The frequency domain beamformer relies on the cross-spectral density matrics. This is calculated for pre and post-stimulus onset data, as well as both data segments together (lines 87–95 in ‘*ASSR_dics.m*’). The latter approach is required for the computation of ‘common’ spatial filters than can be subsequently applied to the pre- and post-stimulus data separately. This effectively minimizes the possibility of condition differences leading to biased filter estimates. Source analysis is applied from lines 111–136 in *ASSR_dics.m*. One thing to note is that the common spatial filters, computed using the ‘dics’-method, are applied to the individual conditions with the ‘pcc’-method [an acronym for Partial and Canonical Correlation (Schoffelen and Gross, 2009)]. This change of method is needed because we used dipole pairs as source models. Although the ‘dics’ and ‘pcc’ methods implement the exact same mathematical algorithm for the computation of the beamformer spatial filters, the methods differ in their flexibility with which the source reconstructed data can be represented. The data representation of the ‘dics’ methods sticks closely to the description in the original paper (Gross et al., 2001). In the present example, where we express the functional data as a grid point specific coherence coefficient, this would result in each point estimate to reflect the modulation signal phase-locked activity of two dipoles at once, which is difficult to interpret. The ‘pcc’ method allows for more fine-grained post-processing of the source reconstructed activities. More specifically, here it is used to separate at each grid point the ‘scanning dipole’-of-interest’s from the ‘suppression dipole’-of-no-interest’s activity, before the computation of the coherence coefficient between the scanning dipole and the modulation signal.

The source analysis outcome is illustrated in **Figure 4** bottom row. Instead of visualizing N1 source power, the largest coherence value with a surrogate 40 Hz signal during the ASSR stimulation period (1–2 s) relative to pre-stimulation baseline (-10) is illustrated. For comparison purposes the hairlines are kept at the same coordinates for both source visualizations (e.g., N1 and 40 Hz coherence). Loading, grand averaging and visualization are performed on lines 166–197 in *ASSR_dics.m* and require 17 s to compute.

### Data Access

The complete dataset, including raw and preprocessed data, as well as all analysis scripts are published and available for download on https://doi.org/11633/di.dccn.DSC_3015000.00_810.

## Results

Analysis in the time-domain revealed a reliable mid-latency ERP components (N1/P2) followed by steady evoked activity (**Figures 1A,B**). The scalp topography of the N1/P2 components shows an extremum at central electrodes, which reversed its polarity between the N1 and P2 component. Both are commonly observed and frequently reported manifestation of evoked activity in the EEG, elicited by the onset of an auditory stimulus.

There was significant difference between pre- and post-stimulus brain activity (**Figures 1D–F**), as confirmed by the cluster-based permutation procedure controlling for multiple comparisons across the spatial (i.e., electrodes) and temporal dimensions. Besides plotting the raw effect size in mV, the scalp topography of the N1 and P2 effect size can be visualized as *t*-values. This allows the strength of the observed effect to be transferred to and expressed as a *Cohen’s d* of 1.85 for *t*-values of 5^5^. Furthermore, time and electrode clusters are visualized reflecting the latencies/electrodes on the basis of which the null hypothesis pertaining to the exchangeability of the pre and post-stimulus data is rejected (**Figures 1D,E**).

Analysis in the frequency domain revealed a prominent 40 Hz peak in the spectrum of the ERP, in accordance with the stimulation frequency (**Figures 2A,B**). Statistical evaluation in the frequency domain confirmed a significant difference in 40 Hz response (**Figure 2C**) with similar effect size as compared to the time domain evaluation.

Combined time and frequency analysis confirmed the steady increase in 40 Hz power, lasting throughout the entire stimulation interval (**Figure 3**). There was a significant difference in 40 Hz power between pre and post-stimulus activation (**Figure 3C**) when controlling for multiple comparisons across the time, frequency and space dimensions.

Finally, source space analysis confirmed the primary generators of the N1 auditory evoked response in the right auditory cortex. This is in line with the experimental condition of left-ear stimulation, reflecting a stronger contra lateral auditory cortex response.

## Discussion

In the present work we build up and document a FieldTrip-based analysis pipeline, starting from single subject and continuing with various types of group-level analyses. To this end, we analyzed EEG data, which was recorded during a common paradigm, the auditory stead state evoked response (ASSR).

The time-domain analysis at the electrode level focused on various aspects of descriptive data evaluation, in time and space (**Figures 1A–C**). This was followed by an inferential statistical procedure, within the framework of a non-parametric permutation test that controls the family-wise error rate using clusters (**Figures 1D–F**), where we evaluated the N1/P2 components in the ERP, relative to a pre-stimulus baseline. Next, a frequency domain analysis revealed a reliable 40 Hz ASSR response (**Figures 2A,B**), which was significantly increased with respect to baseline (**Figure 2C**). Combining time and frequency domains it is also possible to demonstrate how the spectral aspects of the data evolve over time (**Figure 3**). In the example experimental manipulation and dataset these analyses are somewhat redundant, but we included them here for completeness. Typically, the analysis strategy is predominantly motivated by the research question at hand. Thus, in the present case it would suffice to reject the null hypothesis of the exchangeability of the brain responses during pre and post-stimulus periods. However, frequency-domain analyses are inappropriate for the evaluation of mid latencies evoked potentials, as are time-domain analyses when spectral changes over time are anticipated. Here we demonstrated analysis strategies addressing both temporal and spectral aspects of the data.

It should be noted that the statistical control using the probability distribution of a cluster-based test statistic, obtained with a randomization approach does not allow strong statistically motivated inferences about specific temporal, spectral or spatial properties of the data. This inferential procedure tests a null hypothesis of exchangeability of the data, and not a null hypothesis about some specific temporal, spectral or spatial parameter of the data. Instead, these temporal, spectral or spatial properties can be described on the basis of prior knowledge and/or after statistical evaluation using visual inspection of the observed difference in the data. Furthermore, source analysis can be used to estimate the difference in the distribution of the underlying neural generators (**Figure 4**).

In summary, group analyses in time frequency, electrode and source space are readily accessible using the MATLAB-based FieldTrip toolbox. All of the reported analyses are available in various other experimental contexts and are documented on the wiki. Detailed information about getting started with the toolbox is documented here: http://www.fieldtriptoolbox.org/getting_started. Extensive treatment and preprocessing of time domain data can be found here http://www.fieldtriptoolbox.org/tutorial#preprocessing and plethora of frequency and time frequency analyses on scalp level are documented^6^. Decisions under uncertainty in the context of psychophysiological research are covered in several dedicated statistic tutorials http://www.fieldtriptoolbox.org/tutorial#statistics. Preparation and analysis steps during source reconstruction are addressed in extensive detail (see text footnote 4).

## Author Contributions

TP and JS analyzed the data. TP, JS, and RO wrote the manuscript.

## Conflict of Interest Statement

The authors declare that the research was conducted in the absence of any commercial or financial relationships that could be construed as a potential conflict of interest.

## Footnotes

1 http://www.fieldtriptoolbox.org/tutorial/layout

2 http://www.fieldtriptoolbox.org/tutorial/cluster_permutation_timelock

3 http://www.fieldtriptoolbox.org/tutorial/timefrequencyanalysis

4 http://www.fieldtriptoolbox.org/tutorial#source_reconstruction

5 Cohen’s d is calculated using the formula *d* = (t^∗^2)/(sqrt(df)).

6 http://www.fieldtriptoolbox.org/tutorial#sensor-level_analyses
